# A Multi-Omics Integration Framework with Automated Machine Learning Identifies Peripheral Immune-Coagulation Biomarkers for Schizophrenia Risk Stratification

**DOI:** 10.3390/ijms26157640

**Published:** 2025-08-07

**Authors:** Feitong Hong, Qiuming Chen, Xinwei Luo, Sijia Xie, Yijie Wei, Xiaolong Li, Kexin Li, Benjamin Lebeau, Crystal Ling, Fuying Dao, Hao Lin, Lixia Tang, Mi Yang, Hao Lv

**Affiliations:** 1 The Clinical Hospital of Chengdu Brain Science Institute, School of Life Science and Technology, University of Electronic Science and Technology of China, Chengdu 610054, China; 202421140509@std.uestc.edu.cn (F.H.); 202422140207@std.uestc.edu.cn (Q.C.); 202421140508@std.uestc.edu.cn (X.L.); 202421140512@std.uestc.edu.cn (S.X.); 202421140421@std.uestc.edu.cn (Y.W.); 202421140527@std.uestc.edu.cn (X.L.); 202421140412@std.uestc.edu.cn (K.L.); hlin@uestc.edu.cn (H.L.); lixiatang@uestc.edu.cn (L.T.); 2 School of Biological Sciences, Nanyang Technological University, Singapore 639798, Singapore; benjamin.lebeau@ntu.edu.sg (B.L.); crystal.ling@ntu.edu.sg (C.L.); fuying.dao@ntu.edu.sg (F.D.)

**Keywords:** schizophrenia, multi-omics, AI, molecular biomarker, immune–thrombotic dysregulation

## Abstract

Schizophrenia (SCZ) is a complex psychiatric disorder with heterogeneous molecular underpinnings that remain poorly resolved by conventional single-omics approaches, limiting biomarker discovery and mechanistic insights. To address this gap, we applied an artificial intelligence (AI)-driven multi-omics framework to an open access dataset comprising plasma proteomics, post-translational modifications (PTMs), and metabolomics to systematically dissect SCZ pathophysiology. In a cohort of 104 individuals, comparative analysis of 17 machine learning models revealed that multi-omics integration significantly enhanced classification performance, reaching a maximum AUC of 0.9727 (95% CI: 0.8889–1.000) using LightGBMXT, compared to 0.9636 (95% CI: 0.8636–1.0000) with CNNBiLSTM for proteomics alone. Interpretable feature prioritization identified carbamylation at immunoglobulin-constant region sites IGKC_K20 and IGHG1_K8, alongside oxidation of coagulation factor F10 at residue M8, as key discriminative molecular events. Functional analyses identified significantly enriched pathways including complement activation, platelet signaling, and gut microbiota-associated metabolism. Protein interaction networks further implicated coagulation factors F2, F10, and PLG, as well as complement regulators CFI and C9, as central molecular hubs. The clustering of these molecules highlights a potential axis linking immune activation, blood coagulation, and tissue homeostasis, biological domains increasingly recognized in psychiatric disorders. These results implicate immune–thrombotic dysregulation as a critical component of SCZ pathology, with PTMs of immune proteins serving as quantifiable disease indicators. Our work delineates a robust computational strategy for multi-omics integration into psychiatric research, offering biomarker candidates that warrant further validation for diagnostic and therapeutic applications.

## 1. Introduction

Schizophrenia (SCZ) is a complex and heterogeneous psychiatric disorder characterized by disruptions in thought processes, as well as disconnections between emotion and behavior [1,2,3]. Elucidating the underlying pathogenic mechanisms of SCZ is crucial for improving diagnosis, treatment, and patient prognosis. In recent years, advances in sequencing technologies have significantly contributed to the identification of SCZ-related biomarkers. For example, genome-wide association studies (GWASs) have identified multiple risk genes and loci associated with SCZ, including genes involved in synaptic function, neurodevelopment, and ion channel regulation [4]. However, despite these valuable insights, clinical translation remains challenging. Single-omics approaches offer limited power in comprehensively characterizing the molecular landscape of SCZ, highlighting the need for a more integrative strategy [5,6,7,8]. Consequently, identifying multidimensional molecular features of SCZ may pave the way for more targeted and effective therapeutic interventions.

Transcriptomic analysis has emerged as an essential tool for investigating the molecular basis of SCZ. By analyzing gene expression profiles associated with the disorder, researchers can identify key genes and pathways involved in SCZ pathogenesis, as well as potential genetic variants contributing to disease susceptibility and progression [9,10,11]. Recent advancements in artificial intelligence (AI) have further expanded the scope of biomedical research, particularly through machine learning applications for disease feature identification [12,13]. Several studies have applied machine learning models to transcriptomic data from peripheral blood and the prefrontal cortex (PFC) to distinguish SCZ patients from healthy controls [14,15,16,17]. However, these studies face notable limitations, including a lack of external validation for identified genes and insufficient functional analyses, which undermine reproducibility and limit their utility as stable disease response features. Additionally, these studies are predominantly restricted to transcriptomics, lacking the integration of metabolomics, proteomics, epigenomics, etc. This gap underscores the necessity of employing machine learning to integrate multi-omics data to identify more robust disease response signatures and reliable peripheral biomarkers.

Based on these insights, several studies have explored the potential of multi-omics strategies for SCZ biomarker identification and disease classification. For instance, Fan et al. [18] integrated metagenomics, metabolomics, and proteomics data and identified seven serum metabolites significantly associated with cytokines and gut microbiome α-diversity using a random forest approach. Their model achieved an impressive 84.0% average classification accuracy in distinguishing SCZ cases from controls. Additionally, Campeau et al. [19] performed untargeted mass spectrometry analysis of proteomic and metabolomic profiles in SCZ patients and healthy controls, revealing extensive associations between SCZ and dysregulated inflammatory and metabolic systems. However, these studies have yet to fully leverage advanced AI methodologies to capture the comprehensive molecular landscape of SCZ.

**Figure 1:**
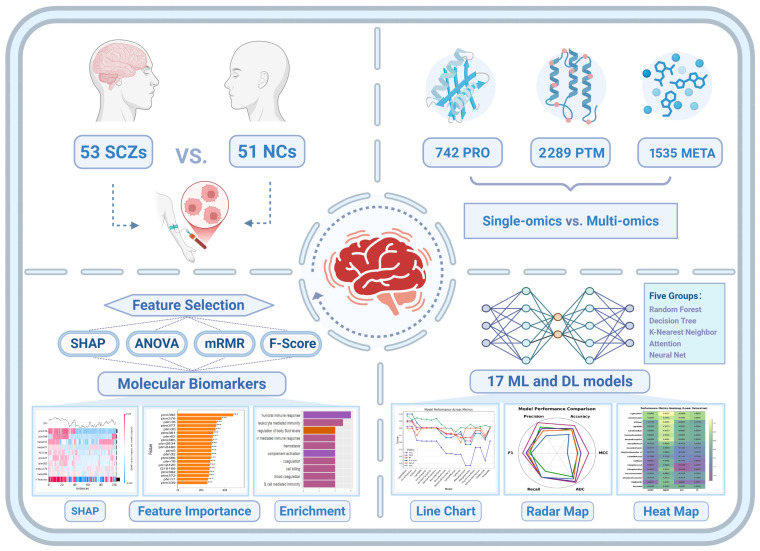
Overview of the integrated multi-omics and AI framework for SCZ classification and biomarker discovery. This study integrates plasma proteomics, PTMs, and metabolomics from 53 SCZ patients and 51 NC subjects to systematically evaluate the classification performance of single-omics versus multi-omics approaches. The machine learning and deep learning framework includes 17 models spanning tree-based, distance-based, and neural network architectures to assess predictive performance across different omics layers. Feature selection was performed using SHAP, ANOVA, mRMR, and F-Score to identify key molecular biomarkers, followed by enrichment and pathway analysis to elucidate biological mechanisms. Visual summaries of feature importance, enrichment results, and classification performance metrics further illustrate the robustness of the multi-omics approach in improving SCZ classification and identifying biologically meaningful molecular signatures. Feature importance with statistical significance are indicated by asterisks, *** *p* < 0.001.

To fill this gap, this study employs an AI-driven multi-omics framework to systematically analyze plasma proteomics, post-translational modifications (PTMs), and untargeted metabolomics data from SCZ patients and non-psychiatric comparison (NC) subjects. Utilizing 17 machine learning models, we constructed classification models and identified key molecular features associated with SCZ. Our results demonstrate that multi-omics integration significantly enhances classification performance, surpassing single-omics approaches in distinguishing SCZ patients from healthy individuals. Notably, our analysis emphasizes immune system dysregulation and coagulation abnormalities as central molecular hallmarks of SCZ. These findings remained consistent across multiple feature selection methods, including Shapley Additive Explanations (SHAPs), Analysis of Variance (ANOVA), Fisher Score (F-score), and Minimum Redundancy Maximum Relevance (mRMR), underscoring their robustness and potential clinical relevance. Taken together, this study not only advances our understanding of the molecular basis of SCZ but also establishes a scalable framework for integrating multi-omics data into psychiatric research. Our findings lay the foundation for future investigations exploring multi-omics biomarkers for early diagnosis, patient stratification, and personalized treatment strategies for SCZ (Figure 1).

## 2. Results

### 2.1. Single-Omics Classification Performance and Model Benchmarking in SCZ

This study utilized publicly available multi-omics data (PMC9054664), which includes three distinct omics layers—plasma proteomics, PTMs, and metabolomics—derived from plasma samples of individuals with SCZ and age-matched NC subjects [19]. A total of 105 individuals, including 54 SCZ patients and 51 NC subjects, were included in the analysis, with quantitative profiling of 742 proteins, 2289 PTMs, and 1535 metabolites obtained using high-resolution mass spectrometry-based approaches. To ensure data integrity and comparability, missing values were imputed using the R (v4.4.3) package *missForest*, followed by rigorous normalization to construct standardized expression profile matrices. Furthermore, only features shared across all three datasets were retained, yielding a harmonized dataset suitable for robust classification analysis.

To systematically model the molecular architecture of SCZ and identify discriminative biomarkers, we employed an integrated machine learning and deep learning framework, combining state-of-the-art ensemble learning methods with customized deep learning architectures. Specifically, we utilized AutoGluon’s automated machine learning pipeline to evaluate a diverse set of machine learning models, including Random Forest, XGBoost, LightGBM, ExtraTrees, KNeighbors, etc., dynamically optimizing hyperparameters and feature selection strategies. Beyond conventional machine learning, we developed four specialized deep learning algorithms—CNNBiLSTM, Transformer, SimpleNN, and AttentionMechanism—designed to capture nonlinear dependencies, hierarchical feature representations, and temporal relationships within high-dimensional omics data. The CNNBiLSTM architecture integrates convolutional feature extraction with bidirectional long short-term memory (BiLSTM) networks to preserve sequential dependencies in molecular profiles. The Transformer model facilitates global context-aware feature learning, leveraging self-attention mechanisms to dynamically assign weights to critical molecular features. SimpleNN serves as a fully connected baseline architecture for rapid classification, while the AttentionMechanism-based model explicitly prioritizes key biomarkers by enhancing feature-level interpretability.

**Figure 2:**
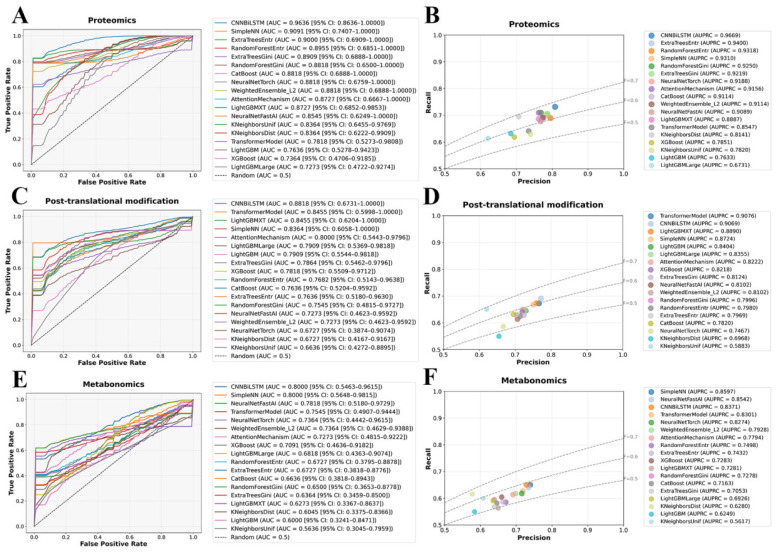
Performance evaluation of single-omics classifiers for SCZ using machine learning and deep learning models. (**A**,**C**,**E**) ROC curves for proteomics, PTMs, and metabolomics based on 17 algorithms, respectively. (**B**,**D**,**F**) PR curves for proteomics, PTMs, and metabolomics based on 17 algorithms, respectively.

To assess the classification performance of different omics layers, we systematically evaluated model performance using Receiver Operating Characteristic (ROC) and Precision–Recall (PR) curves. As shown in Figure 2A,B, plasma proteomics exhibited the highest classification performance, reinforcing the hypothesis that dysregulation at the protein level plays a pivotal role in SZ. CNNBiLSTM achieved the highest area under the curve (AUC = 0.9636 (95% CI: 0.8636–1.0000)), significantly surpassing all traditional machine learning models, highlighting the capacity of deep learning to capture nonlinear molecular interactions. Ensemble models, including SimpleNN and ExtraTreesEntr, also demonstrated strong predictive power (AUC > 0.9000), further establishing the robustness of proteomic markers in distinguishing SZ from healthy controls. PR curve evaluations confirmed this trend, with CNNBiLSTM yielding the highest average precision (AUPRC = 0.9669), followed closely by ExtraTreesEntr (AUPRC = 0.9400) and RandomForestEntr (AUPRC = 0.9318). These results suggest that deep learning architectures can effectively learn hierarchical representations from proteomic features, capturing both local and global patterns indicative of SZ. Conversely, models with limited capacity for handling high-dimensional molecular interactions, such as XGBoost (AUC = 0.7364 (95% CI: 0.4706–0.9185)) and LightGBMLarge (AUC = 0.7273 (95% CI: 0.4722–0.9274)), exhibited the weakest performance, underscoring their inability to effectively model the discriminative power of proteomic signatures.

PTM-based classification exhibited slightly lower but still significant predictive performance, indicating that PTMs encode biologically relevant signatures for SCZ classification, albeit with greater heterogeneity compared to proteomic markers (Figure 2C,D). CNNBiLSTM and Transformer models outperformed traditional machine learning approaches, particularly in PR evaluations, where TransformerModel achieved an AUPRC of 0.9076. This result highlights the suitability of attention-based architectures for extracting meaningful representations from complex PTMs data, where site-specific modifications may introduce higher-order dependencies. The AUC values of CNNBiLSTM (0.8818 (95% CI: 0.6731–1.000)) and TransformerModel (0.8455 (95% CI: 0.5998–1.000)) remained strong, reflecting the ability of deep learning models to capture the regulatory and functional implications of PTMs alterations. In contrast, distance-based models such as KNeighborsDist (AUC = 0.6727 (95% CI: 0.4167–0.9167)) and KNeighborsUnif (AUC = 0.6636 (95% CI: 0.4272–0.8895)) exhibited a more pronounced performance drop, likely reflecting their sensitivity to the high-dimensional and potentially sparse nature of PTMs data. This suggests that PTMs encode relevant molecular alterations but may require specialized feature selection or representation learning approaches to maximize their discriminative potential.

Metabolomics-based classification demonstrated the lowest predictive power among the three omics datasets, which may be attributed to the inherent variability and transient nature of metabolic profiles (Figure 2E,F). Despite these challenges, CNNBiLSTM (AUC = 0.8000 (95% CI: 0.5463–0.9615)) maintained a performance advantage over traditional machine learning models, while SimpleNN (AUPRC = 0.8597) exhibited strong precision-recall trade-offs, suggesting that metabolomic alterations, although more variable, still contain informative patterns that can be leveraged by deep learning. However, decision-tree-based and distance-based models experienced a marked decline in accuracy, with LightGBM (AUC = 0.6000 (95% CI: 0.3241–0.8471)) and KNeighborsUnif (AUC = 0.5636 (95% CI: 0.3045–0.7959)) performing the worst. These results indicate that tree-based and distance-based methods may struggle with the intrinsic complexity and dynamic nature of metabolomic data, where feature redundancy, batch effects, and individual variability introduce significant classification challenges. These findings suggest that additional feature engineering, regularization strategies, or hybrid modeling approaches may be required to extract meaningful predictive signals from metabolomic datasets.

### 2.2. Multi-Omics Integration for Enhanced Classification Performance in SCZ

Existing studies have demonstrated that the pathogenesis of SCZ covers multiple omics levels [20], including genomic [21], proteomic [22], metabolic [23], and PTM regulatory networks [24], each contributing to the intricate molecular architecture of SCZ pathophysiology. While individual omics analyses have provided valuable insights, their inherent limitations in capturing cross-layer biological interactions necessitate a more integrated approach. We hypothesized that a multi-omics strategy, incorporating metabolomics, proteomics, and PTMs, could offer a more comprehensive molecular characterization of SCZ, leading to improved classification performance and enhanced biomarker discovery.

**Figure 3:**
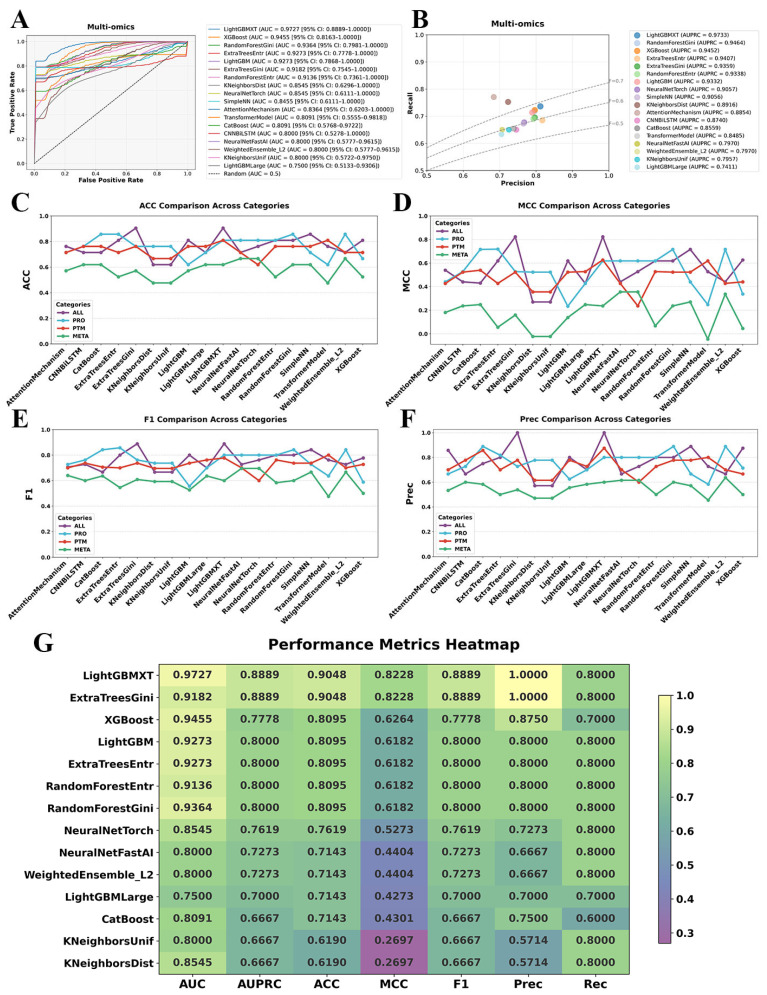
Performance evaluation of multi-omics integration for SCZ classification using machine learning and deep learning models. (**A**) ROC curve of multi-omics integration based on 17 algorithms. (**B**) PR curve of multi-omics integration based on 17 algorithms. (**C**–**F**) Model performance of multi-omics integration in terms of ACC, MCC, F1, and Prec, respectively. (**G**) Heatmap of multi-omics model performance in terms of AUC, AUPRC, ACC, MCC, F1, Prec, and Rec, respectively.

To systematically evaluate the impact of multi-omics integration, we constructed a harmonized dataset by aligning matched plasma proteomics, PTMs, and metabolomics profiles across 104 samples, comprising 53 SCZ patients and 51 NC subjects, along with a total of 4566 features. When leveraging multi-omics dataset for classification modeling, we observed a substantial performance improvement compared to individual omics layers. The integrated multi-omics approach yielded a marked enhancement in discriminative power, with seven models achieving AUC values exceeding 0.9000 (Figure 3A). Notably, the LightGBMXT emerged as the top-performing model, achieving a remarkable AUC of 0.9727 (95% CI: 0.8889–1.000), highlighting its superior ability to leverage the synergistic interactions across different molecular layers. The PR curve analysis further reinforced these findings, demonstrating a noticeable improvement in the precision–recall trade-off for the multi-omics model, underscoring its potential in distinguishing SCZ patients from healthy controls with higher reliability (Figure 3B).

Beyond AUC and PR analysis, we systematically compared additional key evaluation metrics, including accuracy (ACC), Matthews correlation coefficient (MCC), Precision (Prec), and F1 score (F1), across different model categories (Figure 3C–F). The multi-omics models consistently outperformed their single-omics counterparts, demonstrating higher classification accuracy and improved model robustness across diverse algorithmic architectures. Importantly, the multi-omics approach not only enhanced classification performance but also provided greater resilience across different machine learning paradigms, as reflected in its superior MCC scores. These results highlight the ability of integrated omics data to mitigate the limitations of individual molecular layers by capturing complex biological interactions that underlie SCZ pathophysiology.

Figure 3G further consolidates the performance metrics of multi-omics models through a comprehensive heatmap, intuitively summarizing their comparative effectiveness across key indicators, including AUC, AUPRC, ACC, MCC, F1, Prec, and Rec. The heatmap reveals that gradient boosting methods, particularly LightGBMXT, consistently outperformed most other models in AUC and Prec, underscoring its robustness when applied to multi-omics datasets. This observation suggests that gradient boosting techniques are particularly effective in handling the high-dimensional and heterogeneous nature of integrated omics data for binary classification tasks.

The observed performance gains underscore the intrinsic value of multi-omics integration in psychiatric biomarker discovery. While single-omics analyses are constrained by their inability to capture cross-modal interactions, the multi-omics framework leverages complementary biological information across molecular domains, enhancing the interpretability and generalizability of predictive models. Furthermore, deep learning architectures such as CNNBiLSTM and Transformer models demonstrated a heightened ability to extract meaningful representations from the integrated dataset, reinforcing the importance of hierarchical feature learning in deciphering multi-layered biological systems.

### 2.3. Interpretable Multi-Omics Feature Selection Reveals Immune and Coagulation Dysregulation in SCZ

To enhance the interpretability of our classification model, we utilized SHAP to assess the contribution of individual molecular features to SCZ classification. By leveraging SHAP values within the LightGBMXT model, we identified key biomolecular signatures that differentiate SCZ patients from NC subjects, providing insights into the underlying disease mechanisms. Notably, our analysis highlighted features primarily related to immune response and coagulation processes, with specific proteins exhibiting PTMs such as carbamylation and oxidation, which may be functionally relevant to SCZ pathology (Supplementary Tables S1 and S2).

**Figure 4:**
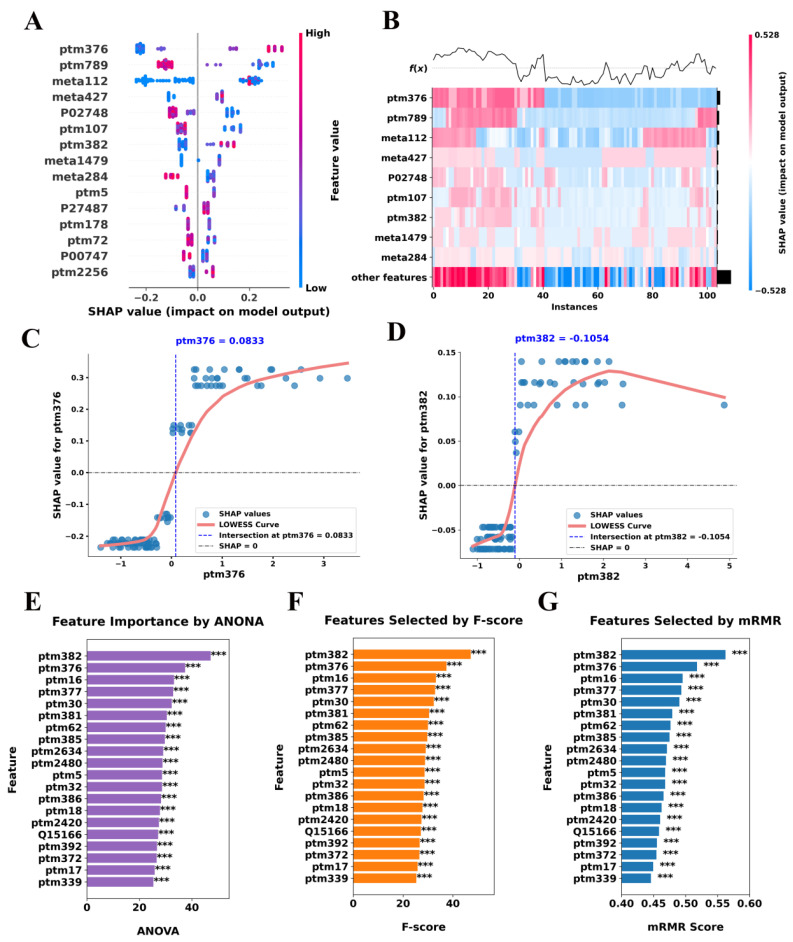
Evaluation and comparison of feature selection across multi-omics approaches using SHAP, ANOVA, F-score, and mRMR. (**A**) A visualization of the impact of different features on the model output, ordered by the magnitude of their SHAP values. The plot illustrates the direction and magnitude of each feature’s impact on the model output, with positive SHAP values indicating an increase in the model’s prediction and negative values indicating a decrease. (**B**) The heatmap depicting the distribution of SHAP values across 100 instances, highlighting the correlation between feature values and their effect on the model’s output. (**C**,**D**) SHAP dependence plots for selected features, ptm376 (**C**) and ptm382 (**D**), demonstrating the relationship between feature values and SHAP values, with intersection points marking significant thresholds for model impact. (**E**–**G**) Feature importance determined by different selection methods: (**E**) ANOVA, (**F**) F-score, and (**G**) mRMR scores. Features are ranked based on their importance, with statistical significance indicated by asterisks. *** *p* < 0.001.

The SHAP summary plot ranks features based on their overall impact on the classification model, revealing that ptm376, ptm789, meta112, and meta427 exerted the strongest influence on model predictions (Figure 4A). Positive SHAP values indicate a direct contribution to SCZ classification, whereas negative values suggest a protective or neutralizing effect. Notably, these key features represent immune- and coagulation-related molecules, further supporting their relevance in SCZ pathophysiology. Importantly, the heatmap in Figure 4B provides a finer resolution of SHAP value distributions across individual samples, demonstrating distinct molecular patterns between SCZ and NC subjects. These findings reinforce the hypothesis that dysregulation of immune and coagulation-related molecules plays a significant role in the molecular landscape of SCZ.

In addition, a particularly striking observation emerges from the SHAP dependence plots, which reveal nonlinear threshold effects in key features (Figure 4C,D). For ptm376 with gene name IGKC, SHAP values exhibit a sharp inflection at approximately 0.0833, suggesting a critical expression threshold beyond which this feature markedly enhances SCZ classification. This transition suggests that low expression levels of ptm376 contribute minimally to model decisions, whereas exceeding the threshold induces a disproportionately large impact on classification probability. This behavior may reflect a functional tipping point in immune or coagulation signaling, warranting further biological investigation. Similarly, ptm382 (with gene name IGHG1) displays a saturation effect at SHAP = −0.1054, indicating that as expression levels increase beyond this threshold, their contribution to model predictions stabilizes rather than continuing to grow linearly. This pattern suggests a potential upper limit in the biological relevance of ptm382 modifications in SCZ, where exceeding a certain expression level does not further alter disease risk. The identification of such threshold-dependent relationships provides a refined molecular stratification framework, suggesting that discrete biomarker cutoffs may be more biologically meaningful than continuous expression changes.

To validate the robustness of our SHAP-based feature selection, we employed complementary analyses using ANOVA, F-score, and mRMR (Figure 4E–G). Remarkably, these methods consistently identified the same immune and coagulation-related features as significant, reinforcing the reliability of our SHAP-based findings (Supplementary Table S1). Moreover, two types of PTMs, with gene names IGKC (P01834) and IGHG1 (P01857), were consistently found to be highly expressed across all statistical methods. Given the well-documented links between immune dysregulation and psychiatric disorders, these results provide compelling evidence that immune-related protein modifications may serve as potential molecular signatures for SCZ diagnosis and risk stratification [25,26,27].

### 2.4. Enrichment Analysis and Protein–Protein Interaction (PPI) Network Analysis of Key Features

To gain deeper insights into the molecular underpinnings of SCZ, we performed functional analyses on the 16 features identified by SHAP. These features were subjected to Gene Ontology (GO) analysis, Kyoto Encyclopedia of Genes and Genomes (KEGG) pathway analysis, and PPI network analysis, aiming to elucidate the key molecular mechanisms involved in SCZ pathophysiology.

**Figure 5:**
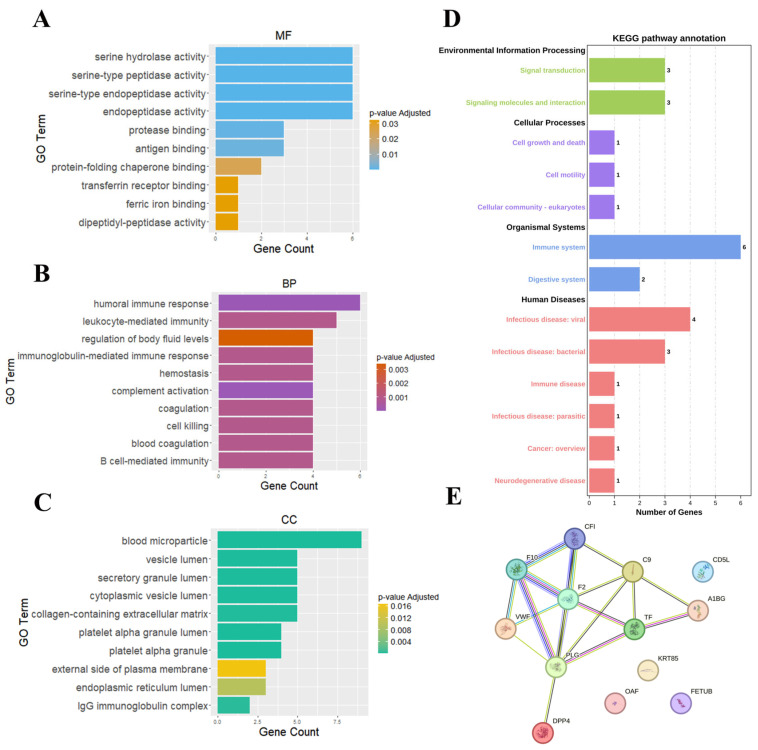
Enrichment analysis and PPI network of key features in SCZ. (**A**–**C**) The GO enrichment analysis of the selected features in terms of MF, BP, and CC, with *p*-values < 0.05 considered significant. (**D**) KEGG pathway analysis reveals significant enrichment in pathways related to environmental information processing, cellular processes, organismal systems, and human diseases. (**E**) PPI network analysis of prioritized proteins derived from multi-omics feature selection.

GO analysis revealed that SCZ-associated features were significantly enriched in immune-related functions, including serine hydrolase activity, serine-type peptidase activity, and antigen binding (Figure 5A). The prominence of these molecular functions (MF) suggests an integral role of proteolytic enzymes and antigen-processing mechanisms in SCZ aligning with the existing evidence that implicates altered immune dysfunction in psychiatric disorders [28,29,30]. In biological processes (BPs), pathways such as humoral immune response, leukocyte-mediated immunity, and complement activation were significantly enriched, reinforced the theory that immune system dysregulation plays a critical role in SCZ (Figure 5B). The interplay between innate and adaptive immune responses, particularly complement system activation, has been increasingly recognized as a key contributor to neuroinflammatory processes underlying SCZ pathogenesis [31,32]. Cellular component (CC) enrichment further pointed to blood microparticles, vesicle lumen, and platelet alpha granules, suggesting that coagulation-related vesicular trafficking and immune signal transduction may be involved in disease progression (Figure 5C).

KEGG pathway analysis highlighted multiple pathways associated with immune system dysfunction, coagulation cascades, and cellular signaling abnormalities (Figure 5D). Notably, the involvement of signal transduction and signaling molecule interactions suggests that dysregulated immune communication may contribute to aberrant neuronal function and inflammation in SCZ. Additionally, cell growth and death, cell motility, and platelet function were enriched, indicating that immune cell migration and platelet activation may play a role in sustaining a chronic pro-inflammatory state in SCZ. The enrichment of immune system pathways, in particular, supports the notion that both innate and adaptive immune mechanisms are implicated in SCZ, potentially through excessive or aberrant immune activation. Furthermore, the enrichment of infectious diseases and immune diseases pathways reinforces the hypothesis that environmental triggers, such as infections or other stressors, may act as modulators of SCZ risk, either through direct neuroimmune interactions or via systemic inflammatory responses. This is particularly relevant in the context of the gut–brain axis, where emerging evidence suggests that microbial metabolites and immune interactions within the gastrointestinal system can modulate neuroinflammation and neurotransmitter homeostasis in psychiatric disorders [18]. The presence of digestive system pathways further supports the hypothesis that gut dysbiosis may contribute to SCZ through immune-mediated mechanisms. Additionally, the enrichment of coagulation-related pathways, including those involving fibrinogen and platelets, suggests a potential link between thrombosis [26,33], neurovascular dysfunction [34,35,36], and SCZ.

The PPI network analysis further substantiated these findings, identifying several key proteins involved in immune responses and coagulation (Figure 5E). Core network hubs included CF1 (Complement factor I), F2 (Prothrombin), F10 (Coagulation factor X), and PLG (Plasminogen), highlighting the intricate link between coagulation and immune pathways. The presence of IGKC and IGHG1, which undergo PTMs such as carbamylation and oxidation, suggests that PTM-driven immune alterations may influence SCZ pathology. PTMs have been increasingly recognized as critical regulators of protein function, and their dysregulation may exacerbate immune system imbalances in SCZ. Notably, components of the complement and coagulation cascades were interconnected within the PPI network, supporting a mechanistic model in which these pathways interact to modulate neuroinflammation and potentially impact blood–brain barrier integrity.

## 3. Discussion

This study leveraged multi-omics data and machine learning models to identify key molecular features associated with SCZ. The findings underscore the advantages of multi-omics integration in enhancing classification performance and provide mechanistic insights into the critical role of immune and coagulation-related processes in the pathophysiology of SCZ.

Our results demonstrate that integrating plasma proteomics, PTMs, and metabolomics significantly improves classification accuracy compared to single-omics approaches. The multi-omics binary classification model achieved an outstanding AUC of 0.9727 (95% CI: 0.8889–1.000), particularly when using the LightGBMXT model, which consistently outperformed other models across multiple evaluation metrics. This improvement highlights the importance of incorporating multiple biological layers to capture the molecular complexity of SCZ, a disorder that likely involves interactions among genetics, proteomics, metabolics, and PTMs. Importantly, the superior performance of the integrated dataset over individual omics layers suggests that no single omics dataset is sufficient to comprehensively characterize SCZ at the molecular level. This finding aligns with previous research showing that multi-omics integration enhances the discovery of disease biomarkers and improves the robustness of classification models in complex disorders, including neuropsychiatric diseases [37,38,39,40].

Among the individual omics layers, plasma proteomics exhibited the strongest classification capability, consistent with studies reporting that protein-level alterations are more directly linked to the molecular manifestations of psychiatric disorders [41,42,43]. However, PTMs and metabolomics contributed complementary biological information, and their integration with proteomics further enhanced the discriminatory power of the model. SHAP analysis further identified key immune and coagulation-related molecular features driving model predictions, with PTMs-related proteins, such as IGKC and IGHG1, further suggesting a role for PTMs—particularly oxidation and carbamylation—in immune system dysfunction. These findings are supported by previous studies linking PTM dysregulation to immune-related abnormalities in SCZ [44,45].

Functional enrichment and PPI network analyses further corroborated the involvement of immune and coagulation pathways in SCZ pathophysiology. GO and KEGG pathway analyses revealed significant associations with immune system processes, including humoral immune response, complement activation, and leukocyte-mediated immunity. These results support accumulating evidence that SCZ is characterized by systemic immune dysregulation and chronic low-grade inflammation, which contribute to disease onset and progression [31,32,46,47,48,49,50,51,52]. In addition to immune pathways, KEGG enrichment highlighted coagulation-related processes, reinforcing the growing recognition of vascular dysfunction in SCZ. Several coagulation factors, including F1, F2, F10, and PLG, were central nodes in the PPI network, suggesting that immune-coagulation crosstalk may play a previously underappreciated role in SCZ pathology. This aligns with recent findings implicating hypercoagulation and platelet dysfunction in neuroinflammation and psychiatric disorders [53,54,55].

An intriguing observation from the KEGG analysis was the enrichment of pathways associated with the gut–brain axis, including microbial metabolism and host immune interactions. Recent studies have highlighted the role of gut microbiota in modulating neuroinflammatory responses and neurotransmitter signaling, suggesting that microbiome-derived metabolites may influence SCZ pathophysiology [56,57,58]. Our findings add to this growing body of evidence indicating that gut–immune interactions could be a contributing factor in SCZ, possibly through systemic inflammation or metabolic disruptions. Given the increasing recognition of the microbiome’s role in psychiatric disorders, future studies should explore its interplay with immune dysfunction in SCZ, particularly through integrative metagenomic and metabolomic approaches.

Importantly, our findings both reinforce and expand upon those reported in the original study, which analyzed the same dataset using classical statistical methods [19]. Their analysis emphasized immune and inflammatory dysregulation, including complement and acute-phase pathways—core features that our models also identified through SHAP-based interpretability methods. However, our AI-based integration approach uncovered additional discriminative features, including post-translational modifications such as oxidation of F10 at M8 and carbamylation at IGKC_K20, which were not prominently discussed in the original report. Moreover, while Campeau et al. utilized univariate testing and pathway enrichment analysis, our deep learning models captured nonlinear interactions across omics layers, generating interpretable feature rankings through SHAP values. This distinction in analytical framework allowed us to uncover additional multivariate signals and propose new hypotheses for further biological validation.

These findings have significant implications for biomarker discovery and therapeutic development in SCZ. The identification of immune and coagulation-related molecular features provides a strong rationale for further investigating inflammation-targeted interventions in SCZ. Emerging evidence suggests that immunomodulatory therapies, including anti-inflammatory agents and cytokine-targeting drugs, may hold promise for SCZ treatment [32,59]. Moreover, our results highlight the potential utility of PTM-based biomarkers for disease stratification, as PTMs often reflect dynamic pathological changes that are more sensitive indicators of disease state than static genetic markers [60,61]. Future work should validate these biomarkers in larger cohorts and assess their clinical applicability for early diagnosis and personalized treatment strategies.

While this study provides valuable insights into the molecular underpinnings of SCZ, further validation in independent cohorts and expanded datasets will be beneficial to strengthen the generalizability of our findings. Additionally, integrating additional layers of omics data, such as genomics and epigenomics, could further refine the mechanistic understanding of SCZ. Future studies should aim to build upon this framework by incorporating longitudinal data and expanding the scope of multi-omics integration to uncover dynamic changes in disease progression.

In conclusion, our study highlights the power of multi-omics integration in elucidating the molecular underpinnings of SCZ, demonstrating that immune and coagulation pathways play a crucial role in the disorder’s pathophysiology. The incorporation of advanced machine learning models not only improved classification accuracy but also facilitated the identification of novel molecular features that may serve as potential biomarkers. Moving forward, efforts should focus on validating these findings in larger, multi-ethnic populations and exploring therapeutic strategies targeting immune and coagulation dysfunction in SCZ.

## 4. Materials and Methods

### 4.1. Data Collection

This study utilized publicly available multi-omics data (PMC9054664), which includes plasma proteomics, PTMs, and metabolomics data from 105 individuals, consisting of 54 SCZ patients and 51 age-matched NC subjects. The data were obtained using mass spectrometry-based approaches, allowing for the quantification of 742 proteins, 2289 PTMs, and 1535 metabolites.

### 4.2. Preprocessing and Data Integration

To ensure consistency and facilitate robust analysis, only the features that were common across all three omics datasets were retained. The data were preprocessed to address missing values, utilizing the *missForest* imputation method in R (v4.4.3). The final datasets were standardized to ensure comparability across the omics layers and retained only those samples that were common across all three datasets. Data integration was performed by aligning the samples across the three omics layers (proteomics, PTMs, and metabolomics) while maintaining the integrity of each individual feature set. This integration resulted in a combined dataset consisting of 104 samples, including 53 SCZ patients and 51 NC subjects, with a total of 4566 features.

### 4.3. Machine Learning and Model Construction

We employed a diverse range of machine learning models to analyze the integrated multi-omics dataset. A total of 17 classification algorithms were applied, which included tree-based (e.g., RandomForestGini, ExtraTreesGini), gradient boosting-based (e.g., LightGBM, XGBoost), neural networks (e.g., NeuralNetTorch, CNNBiLSTM), attention-based (e.g., Transformer, AttentionMechanism), and k-nearest neighbors (e.g., KNeighborsUnif, KNeighborsDist). The AutoGluon library was utilized to implement built-in models, and four custom deep learning algorithms were also used for binary classification of SCZ patients versus healthy controls. The dataset was split into training and test sets using an 8:2 ratio, where 80% of the data was used for training and 20% for testing to ensure independent and unbiased performance evaluation. The performance of these models was evaluated using multiple metrics, including AUC, AUPRC, ACC, MCC, F1, Prec, and Rec.

#### 4.3.1. AutoGluon

AutoGluon is an open-source AutoML framework designed to automate the machine learning pipeline for tabular datasets. It simplifies model training, hyperparameter optimization, and evaluation, making it ideal for both beginners and experts. AutoGluon supports various models, including tree-based methods (e.g., LightGBM, XGBoost), neural networks, and k-nearest neighbors, and automatically handles data preprocessing, model selection, and cross-validation to avoid overfitting. Its ability to handle different feature types and optimize models with minimal user input makes it an efficient tool for quick and high-performance machine learning model construction.

#### 4.3.2. Transformer Model

The Transformer model used in this study is based on the Transformer encoder architecture, designed to process sequential input data through self-attention mechanisms. The model is defined as follows:

(1) output = sigmoid(fc(Transformer(x)))

where $x\;\in \;R^{\left(batch\_size,input\_dim\right)}$ is the input data, passed through the transformer encoder layer. The transformer mechanism applies attention to compute a weighted sum of input features. The model architecture consists of the following key components:

Transformer Encoder Layer: The self-attention mechanism in the encoder layer computes the attention scores as follows:

(2) $$Attn(Q,K,V)\;=\;softmax(\frac{QK^{T}}{\surd d_{k}})V$$

where Q, K, and V are the query, key, and value matrices derived from the input data, and $d_{k}$ is the dimensionality of the key vectors.

The output from the transformer is passed through a fully connected layer to produce the final binary classification output.

#### 4.3.3. Attention Mechanism

The Attention Mechanism model applies a scaled dot-product attention mechanism to learn the importance of different input features. The architecture of this model is as follows:

(3) output = sigmoid(fc(Attention(x)))

where the attention mechanism computes the output as

(4) $$Attn\_Output=softmax(\frac{QK^{T}}{\surd d_{k}})V$$

where Q, K, and V represent the query, key, and value matrices, respectively, and ddd is the dimensionality of the model. After applying attention, the output is passed through a fully connected layer followed by a dropout layer to avoid overfitting, and the final binary output is generated using the sigmoid activation function.

#### 4.3.4. CNNBiLSTM

The CNNBiLSTM model combines convolutional neural networks (CNNs) for local feature extraction and bidirectional LSTMs for sequential modeling. The output from both networks is concatenated and passed through a fully connected layer. The model is formulated as follows:

(5) output = sigmoid(fc(CNN(x), BiLSTM(x)))

The CNN layer applies convolution to extract local features from the input sequence, followed by ReLU activation. The BiLSTM layer, which captures both forward and backward dependencies in the sequence, is defined as

(6) $$h_{t}=LSTM(x_{t}\;,\;h_{t-1})$$

where $x_{t}$ is the input at time step t, and $h_{t\;-\;1}$ is the previous hidden state. The final concatenated features are then passed through a fully connected layer for classification.

#### 4.3.5. SimpleNN

The SimpleNN model is a basic fully connected neural network with one hidden layer and a ReLU activation function. The model is defined as follows:

(7) output = sigmoid(fc(ReLU(x)))

where x is the input to the hidden layer. The hidden layer is followed by a dropout layer, which helps prevent overfitting by randomly setting a fraction of input units to zero during training. The final output is obtained using the sigmoid activation function, suitable for binary classification tasks.

### 4.4. Evaluation Metrics

In this study, we adopted a range of widely recognized machine learning evaluation metrics, including ACC, MCC, F1, Prec, and Rec. To determine the area under the curve (AUC, AUPRC), the ROC and PR were drawn [62,63,64]. The particular formulas for these measurements are given below:

(8) $$\mathrm{ACC}\;=\;\frac{\mathrm{TP}\;+\;\mathrm{TN}}{\mathrm{TP}\;+\;\mathrm{FP}\;+\;\mathrm{TN}\;+\;\mathrm{FN}}$$

(9) $$\mathrm{MCC}=\frac{\left(\mathrm{TP}\;\times \;\mathrm{TN}\right)-\left(\mathrm{FP}\;\times \;\mathrm{FN}\right)}{\sqrt{\left(\mathrm{TP}+\mathrm{FP}\right)\left(\mathrm{TP}+\mathrm{FN}\right)\left(\mathrm{TN}+\mathrm{FP}\right)\left(\mathrm{TN}+\mathrm{FN}\right)}}$$

(10) $$\mathrm{Prec}=\frac{\mathrm{TP}}{\mathrm{TP}+\mathrm{FP}}$$

(11) $$\mathrm{Rec}=\frac{\mathrm{TP}}{\mathrm{TP}+\mathrm{FN}}$$

where TP, TN, FP, and FN represent the number of true positives, true negatives, false positives, and false negatives, respectively.

### 4.5. Feature Selection and Interpretability

Feature selection was performed using SHAP to enhance the interpretability of the most promising model, LightGBMXT, and to identify molecular features that significantly contributed to model predictions. SHAP values were used to assess the impact of individual features on the classification results, allowing us to pinpoint key proteins and PTMs associated with SCZ. In addition to SHAP, other statistical methods for feature selection, including ANOVA, F-score, and mRMR, were employed to validate the robustness of the selected features.

#### 4.5.1. SHAP

SHAP is a powerful tool for explaining individual predictions by assigning each feature a contribution to the model’s output [65,66]. It is based on Shapley values, originally derived from cooperative game theory. The Shapley value for a feature represents its average contribution across all possible combinations of features.

For a given prediction, the Shapley value $\varphi_{i}$ for feature i is computed as

(12) $$\varphi_{i}(f)=\sum_{S\subseteq N\backslash \{i\}}\frac{|S|!(|N|-|S|-1)!}{|N|!}[f(S\cup \{i\})-f(S)]$$

where f(S) is the model prediction using the set of features S; N is the set of all features, and $\varphi_{i}(f)$ represents the feature i’s Shapley value.

SHAP values provide a way to evaluate the importance of each feature by summing its contribution across all subsets of the data. Features with higher SHAP values are deemed more important.

#### 4.5.2. ANOVA

ANOVA is a statistical method used to compare the means of different groups to determine whether there is a statistically significant difference between them [67]. In feature selection, ANOVA can be used to identify which features have the most significant variance with respect to the target variable.

For a given feature $X_{i}$, ANOVA computes the *F*-statistic as

(13) $$F=\frac{Between-group\;variance}{Within-group\;variance}=\frac{\frac{1}{k}\sum_{j=1}^{k}n_{j}{\left({\bar{X}}_{j}-\bar{X}\right)}^{2}}{\sum_{i=1}^{k}{\left(X_{i}-\bar{X}\right)}^{2}}$$

where k is the number of groups; $n_{j}$ is the number of samples in group j; ${\bar{X}}_{j}$ is the mean of group j; and $\bar{X}$ is the overall mean.

A higher F-value indicates that the feature is more significant, meaning it has greater explanatory power in relation to the target variable. Features with higher F-values are retained for further model training.

#### 4.5.3. mRMR

The mRMR method selects features that have the highest relevance to the target variable while ensuring minimal redundancy among the features [68]. It aims to maximize the mutual information between each feature and the target while minimizing the pairwise mutual information between the features.

The mRMR criterion is defined as

(14) $$mRMR(S)=\sum_{i\in S}I(X_{i}\;,\;Y)-\frac{1}{{\left|S\right|}^{2}}\sum_{i,j\in S}I(X_{i}\;,\;Xj)$$

where $I(X_{i}\;,\;Y)$ is the mutual information between feature $X_{i}$ and the target Y; $I(X_{i}\;,\;Xj)$ is the mutual information between two features $X_{i}$ and Xj; and S is the set of selected features.

By maximizing relevance and minimizing redundancy, mRMR selects a subset of features that are highly informative and non-redundant, improving the efficiency of the model without sacrificing predictive performance.

#### 4.5.4. F-Score

The F-score is a statistical test used to evaluate the importance of each feature in classification tasks. It measures the ratio of between-class variance to within-class variance for each feature. Features with higher F-scores are more discriminative, meaning they are better at distinguishing between different classes.

The F-score for a feature $X_{i}$ is computed as

(15) $$F_{i}=\frac{\frac{1}{c}\sum_{c=1}^{C}n_{c}{\left(u_{c}-u\right)}^{2}}{\frac{1}{N}\sum_{i=1}^{N}{\left(X_{i}-u\right)}^{2}}$$

where C is the number of classes; $n_{c}$ is the number of samples in class c; $u_{c}$ is the mean of feature $X_{i}$ in class c; and u is the overall mean of the feature.

A higher F-score indicates a greater ability of the feature to distinguish between classes, making it an important feature for the classification task.

### 4.6. Enrichment and Pathway Analysis

To further investigate the biological significance of the identified molecular features, GO and KEGG pathway analyses were performed. These analyses aimed to provide insights into MF, BP, CC, and signaling pathways implicated in SCZ. Additionally, PPI network analysis was conducted to examine the interactions between key proteins and their role in the immune and coagulation pathways related to SCZ.

### 4.7. Statistical Analysis

All statistical analyses were performed using R (v4.4.3) and Python (v3.12.5), with appropriate packages for preprocessing (e.g., *missForest* for imputation), machine learning model construction (e.g., AutoGluon), and statistical tests (e.g., ANOVA, F-score, mRMR for feature selection). The significance level for all statistical tests was set at *p* < 0.05.

## 5. Conclusions

This study presents a comprehensive AI-driven multi-omics framework that integrates plasma proteomics, PTMs, and metabolomics to uncover molecular signatures associated with SCZ. Our findings demonstrate that multi-omics integration significantly outperforms single-omics approaches in disease classification, with the LightGBMXT model achieving a remarkably high AUC of 0.9727 (95% CI: 0.8889–1.000). Through interpretable machine learning and rigorous statistical validation, we identified immune-related PTMs and coagulation-associated molecular features—particularly modifications in immunoglobulin components IGKC and IGHG1 and coagulation factors F10 and F2—as critical discriminators between SCZ patients and healthy controls.

The enrichment analyses and protein–protein interaction networks reveal a robust immune–thrombotic axis underlying SCZ pathophysiology, offering mechanistic insights that extend beyond conventional neurocentric models. These findings underscore the importance of peripheral molecular interactions in psychiatric disorders and suggest that aberrant immune–coagulation crosstalk may contribute to neuroinflammatory and vascular dysfunctions observed in SCZ. Moreover, the observed relevance of gut-associated metabolic pathways points to a potential role of the gut–brain axis in modulating immune responses and disease risk.

By establishing a scalable, interpretable, and high-performing computational pipeline, this study advances the field of psychiatric biomarker discovery and offers a new paradigm for understanding SCZ as a systemic, multi-organ disorder. The identified molecular features not only serve as promising candidates for peripheral diagnostic biomarkers but also provide a biological rationale for developing targeted immunomodulatory and anti-thrombotic interventions. Future validation in larger and ethnically diverse cohorts, along with expansion to longitudinal and interventional datasets, will be essential to translate these insights into clinical applications.

## Data Availability

The data used in this study are publicly available and were originally generated and published by Campeau et al. (2022) [19]. The proteomics data can be accessed via the ProteomeXchange repository with the identifier PXD024474, and the metabolomics data can be accessed via the MassIVE repository with the identifier MSV000086975. All analysis code and model implementation are openly available at our GitHub repository: https://github.com/totnii52/AI-Driven-Multi-Omics-Dissection-of-Immune-Thrombotic-Dysregulation-in-Schizophrenia. The resources and tools used in our analyses are described in the Methods Section (Section 4).

## Supplementary Materials

The following supporting information can be downloaded at: https://www.mdpi.com/article/10.3390/ijms26157640/s1.
