# Supplementary material for: A Multi-Omics Integration Framework with Automated Machine Learning Identifies Peripheral Immune-Coagulation Biomarkers for Schizophrenia Risk Stratification
Source: Int J Mol Sci. 2025 Aug 7;26(15):7640. doi: 10.3390/ijms26157640 (PMC12347864; doi:10.3390/ijms26157640)
Supplement: Supplementary file 1 [file ijms-26-07640-s001.zip › ijms-3775418-supplementary.pdf]

Supplementary Table S1. Key molecular features identified in SCZ patients through SHAP analysis.

Feature	Gene	Protein	Information
ptm376	IGKC	Immunoglobulin kappa constant	P01834_VDNALQSGNSQESVTEQDSKDSTYSLSSTLTLSK_K20(Carbamyl)
ptm789	F10	Coagulation factor X	P00742_DWAESTLMTQK_M8(Oxidation)
P02748	C9	Complement component C9	
ptm107	CD5L	CD5 antigen-like	O43866_GQWGTVCDDGWDIK_
ptm382	IGHG1	Immunoglobulin heavy constant gamma 1	P01857_ALPAPIEKTISK_K8(Carbamyl)
meta1479			Benzoic acid esters/Benzoic acids and derivatives
ptm5	VWF	von Willebrand factor	P04275_VREIRYQGGNR_R2(Carbamyl)N10(HexNAc(4)Hex(5)Fuc(1))
P27487	DPP4	Dipeptidyl peptidase 4	
ptm178	CFI	Complement factor I	E7ETH0_TMGYQDFADVVCYTQK_
ptm72	F2	Prothrombin	P00734_GHVNITRSGIECQLWR_N4(HexNAc(4)Hex(5)NeuAc(2))
P00747	PLG	Plasminogen	
ptm2256	A1BG	Alpha-1B-glycoprotein	P04217_LELHVDGPPPR_H4(Oxidation)
P78386	KRT85	Keratin, type II cuticular Hb5	
ptm2534	FETUB	Fetuin-B	Q9UGM5_TAECPGPAQNASPLVLPP_
ptm364	TF	Serotransferrin	P02787_KSASDLTWDNLK_K1(Carbamyl)
P19652	ORM2	Alpha-1-acid glycoprotein 2	
Q86UD1	OAF	Out at first protein homolog	

Features selected by SHAP analysis, along with their corresponding gene names, protein names, and PTMs information.

Supplementary Table S2. Key molecular features identified in SCZ patients through ANOVA, F-score, and mRMR Analysis.

Feature	Gene	Protein	Information
ptm382	IGHG1	Immunoglobulin heavy constant gamma 1	P01857_ALPAPIEKTISK_K8(Carbamyl)
ptm376	IGKC	Immunoglobulin kappa constant	P01834_VDNALQSGNSQESVTEQDSKDSTYSLSSTLTLSK_K20(Carbamyl)
ptm16	IGHM	Immunoglobulin heavy constant mu	P01871_YKNNSDISSTR_N3(HexNAc(4)Hex(5)Fuc(1))
ptm377	VTN	Vitronectin	P04004_NNATVHEQVGGPSLTSDLQAQSK_N2(HexNAc(4)Hex(5)NeuAc(1))K23(HCysteinyl)
ptm30	C2	Complement C2	P06681_EVVTDQFLCSGTQEDESPCKGESGGAVFLERR_R31(Dihydroxyimidazolidine)
ptm381	IGHG1	Immunoglobulin heavy constant gamma 1	P01857_VSNKALPAPIEK_K4(Carbamyl)
ptm62	C2	Complement C2	P06681_EVVTDQFLCSGTQEDESPCKGESGGAVFLER_
ptm385	HP	Haptoglobin	P00738_YQCKNYYK_K4(Carbamyl)
ptm2634	A2M	Alpha-2-macroglobulin	P01023_SIYKPGQTVK_K4(Carbamyl)
ptm2480			B4E1Z4_GVTTTPWSLAR_
ptm5	VWF	von Willebrand factor	P04275_VREIRYQGGNR_R2(Carbamyl)N10(HexNAc(4)Hex(5)Fuc(1))
ptm32	KNG1	Kininogen-1	P01042_QVVAGLNFR_
ptm386	IGHG2	Immunoglobulin heavy constant gamma 2	P01859_GLPAPIEKTISK_K8(Carbamyl)
ptm18	A2M	Alpha-2-macroglobulin	P01023_LVHVEEPHTETVR_
ptm2420	RBP4	Retinol-binding protein 4	P02753_LLNNWDVCADMVGTFTDTEDPAK_M11(Oxidation)
Q15166	PON3	Serum paraoxonase/lactonase 3	
ptm392	HP	Haptoglobin	P00738_VMPICLPSKDYAEVGR_K9(Carbamyl)

Features selected by ANOVA, F-score, and mRMR analysis, along with their corresponding gene names, protein names, and PTMs information.
